# Water Droplet Erosion of Wind Turbine Blades: Mechanics, Testing, Modeling and Future Perspectives

**DOI:** 10.3390/ma13010157

**Published:** 2019-12-31

**Authors:** Mohamed Elhadi Ibrahim, Mamoun Medraj

**Affiliations:** Department of Mechanical and Industrial Engineering, Concordia University, 1455 De Maisonneuve Blvd. W., Montreal, QC H3G 1M8, Canada; mo_bra@encs.concordia.ca

**Keywords:** water droplet erosion, leading edge erosion, wind turbine blades, damage mechanisms, erosion testing, erosion prediction models

## Abstract

The problem of erosion due to water droplet impact has been a major concern for several industries for a very long time and it keeps reinventing itself wherever a component rotates or moves at high speed in a hydrometer environment. Recently, and as larger wind turbine blades are used, erosion of the leading edge due to rain droplets impact has become a serious issue. Leading-edge erosion causes a significant loss in aerodynamics efficiency of turbine blades leading to a considerable reduction in annual energy production. This paper reviews the topic of water droplet impact erosion as it emerges in wind turbine blades. A brief background on water droplet erosion and its industrial applications is first presented. Leading-edge erosion of wind turbine is briefly described in terms of materials involved and erosion conditions encountered in the blade. Emphases are then placed on the status quo of understanding the mechanics of water droplet erosion, experimental testing, and erosion prediction models. The main conclusions of this review are as follow. So far, experimental testing efforts have led to establishing a useful but incomplete understanding of the water droplet erosion phenomenon, the effect of different erosion parameters, and a general ranking of materials based on their ability to resist erosion. Techniques for experimentally measuring an objective erosion resistance (or erosion strength) of materials have, however, not yet been developed. In terms of modelling, speculations about the physical processes underlying water droplet erosion and consequently treating the problem from first principles have never reached a state of maturity. Efforts have, therefore, focused on formulating erosion prediction equations depending on a statistical analysis of large erosion tests data and often with a combination of presumed erosion mechanisms such as fatigue. Such prediction models have not reached the stage of generalization. Experimental testing and erosion prediction efforts need to be improved such that a coherent water droplet erosion theory can be established. The need for standardized testing and data representation practices as well as correlations between test data and real in-service erosion also remains urgent.

## 1. Introduction

There has been a growing interest in the utilization of wind energy as a promising sustainable energy source that has low to zero emissions. The European Union (EU) installed 11.7 GW of new wind energy in 2018 [1], and is planning to see new installations of wind energy capacity at an average rate of 17.4 GW a year between 2019 and 2022 [2]. Also, the US added 7.5 GW of new wind capacity in 2018, increasing its combined capacity to 96.4 GW [3]. Globally, installed wind energy in 2017 only was estimated to be 52.6 GW [4], thus increasing the cumulative capacity by nearly 11% to around 539 GW [5]. The trend towards higher wind capacity has created a demand for an increased energy capture per utility-scale wind turbine, which is translated into a rapid increase in the turbine blade length (i.e., turbine diameter) [6]. For example, the EU [2] aims to increase wind turbine capacity to 4 MW+ and 8 MW+ for onshore and offshore, respectively, which would necessitate a rotor diameter of more than 200 m [7]. The large diameters have resulted in high linear speed ranges attained by the tip of the wind turbine blade. The tip speed can reach up to 100 m/s in the existing wind turbines [6]. The increase in the turbine size and the blade tip speed brought about several challenges regarding the operation and maintenance of the wind turbine. Among these challenges, leading edge erosion (LEE) due to rain droplet impact has recently become an industry-wide concern. 

The leading edge erosion (LEE) problem arises when a turbine blade surface interacts with water droplets at high speeds [8]. The primary source of water droplets is rain due to the fact that wind turbines are often subjected to heavy rain conditions, especially in offshore farms [9]. Although LEE was expected to occur mainly after a long time of operation [10], real in-service examples of LEE are reported after a relatively short period of operation. Wittrup [11] reported that 273 blades in Horns Rev 2 offshore wind farm were refurbished. The blades have less than seven years in operation. According to Bech et al. [12], several blades at Anholt offshore wind farm were planned to be dismantled and brought ashore in 2018 after less than five years of operation, particularly for the repair of the leading-edge damage. This has generated an interest in studying leading edge erosion of wind turbine blades.

Although aerospace, steam turbine, and gas turbine industries have recognized and studied water droplet impact erosion phenomenon, the problem is so complex that a satisfying understanding and ways of treatments are still lacking. This paper reviews the topic of water droplet erosion with a focus on leading edge erosion of wind turbine blades. General definitions and important distinctions regarding droplet impact erosion as well as its industrial applications will first be presented. Leading edge erosion of wind turbine blades will then be detailed. Physical understanding of droplet impact, mechanisms of erosion damage, and influence of erosion parameters will be discussed. Rain erosion experimental testing facilities and erosion data representation will then be outlined. Erosion modelling and prediction attempts will also be reviewed. A brief discussion about the current understanding of erosion behaviour of polymeric materials will be explored, followed by a discussion on industrial measures used to protect against LEE. Finally, challenges and future research opportunities will be outlined.

## 2. Water Droplet Erosion: Background and Industrial Applications

Water droplet erosion (WDE) is a form of materials wear that is caused by the impact of liquid droplets with sufficiently high speed [13]. The problem was traditionally known also as liquid impingement erosion (LIE) [14]. Discrete water droplets are emphasized to distinguish the WDE phenomenon from liquid jet erosion and cavitation. The range of impact pressures caused by discrete water droplet impact is considerably higher than the stagnation pressure caused by liquid jet. WDE is distinguished from cavitation erosion in the sense that WDE is usually encountered with gaseous or vaporous phase containing discrete liquid droplets, whereas cavitation erosion is observed when a continuous liquid phase carries within it discrete gaseous bubbles or cavities [14]. 

**Figure 1:**
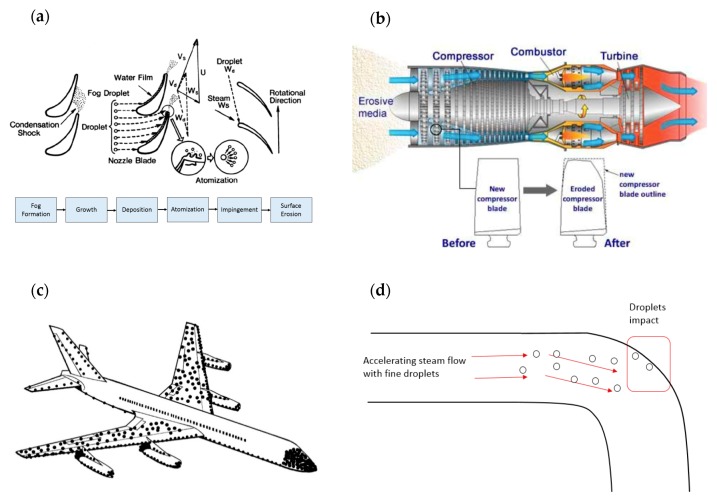
Examples of WDE (wet droplet erosion) occurrence; (**a**) blades of low-pressure stage in steam turbine [32], (**b**) compressor of gas turbine (courtesy of MDS Coatings Technologies Corp.), (**c**) parts of airplane, redrawn from [25], and (**d**) pipes of nuclear power plants [29].

Water droplet erosion (WDE) of solid surfaces has long been a concern for most of high-speed moving components in environments containing water droplets. Blades of several types of machinery seem to be particularly prone to experiencing WDE damage. The problem was first recognized in steam turbine blades in the early 1920s [15,16]. WDE damage is observed on the blades of the low pressure (LP) cycle. This is because steam turbine designers usually aim to maximize the length of the turbine blade at the low-pressure cycle in order to improve the output energy and overall efficiency of the turbine [17]. The increase in the blade length increases the tip speed of the blade to as high as 900 m/s. Such high speed, in the wet steam medium, causes erosion in the blade [18]. Figure 1a illustrates the sequence of the erosion process experienced in a steam turbine blade. WDE is also seen in compressor blades of gas turbines, where the inlet air is cooled (fog cooling) to maximize air density and intake air mass. This will, in turn, increase the power output of the turbine, and therefore, improve the efficiency of the unit [19]. Although this method was proven to be effective, droplets are observed to cause severe damage to the leading edge of the compressor blades [20], as illustrated in Figure 1b. This damage causes vibrations, which in turn increases the loss in the efficiency and results in serious fatigue damages [21]. WDE due to rain erosion is seen also in helicopters, where leading edges of rotor blades are observed to experience significant erosion even at subsonic speeds [22]. More recently, aero-engine fan blades [23] and compressor of turbocharges in automobiles [24] have begun to encounter WDE damage as well. 

WDE is primarily a rotating blades problem, but also seen in linearly moving objects, such as rockets and aeroplanes. For example, erosion caused by raindrop impact of the aircraft surfaces has been an issue in aviation [25,26,27]. Different parts of the aircraft experience different impact intensity and erosion is more pronounced in components made of brittle materials such as glass or thermosetting plastic domes and fibre-reinforced plastic radomes [26]. Erosion damage appears in the form of pitting of airfoils, paint stripping, and/or failure of rivets [26]. Figure 1c shows a schematic of damage intensity distribution across the body of a commercial aeroplane [28]. 

WDE is also observed in carbon steel pipelines used in nuclear/fossil power plants and is known to cause the so-called wall-thinning [29,30,31]. This usually happens when accelerated flow of steam passes through orifices, impinging bent parts of the pipe, as illustrated in Figure 1d. This eventually leads to the leak of the steam flow to the surrounding environment, as it was the case of the Onagawa power plant in 2007 [31]. 

Table 1 provides a summary of the important erosion conditions, namely; the impact speed and droplet size, encountered in different applications. It should be noted that impact speed and droplet size are difficult to quantify for these applications and only approximate ranges could be provided. These values are often concluded after characterization of eroded components, except for rain erosion where the characteristics of raindrops can be studied from meteorological observation. Water droplet erosion encountered in the leading edge of wind turbine blades is detailed in the next section.

## 3. Rain Erosion of Wind Turbine Blade

### 3.1. Blade Design and Material

**Figure 2:**
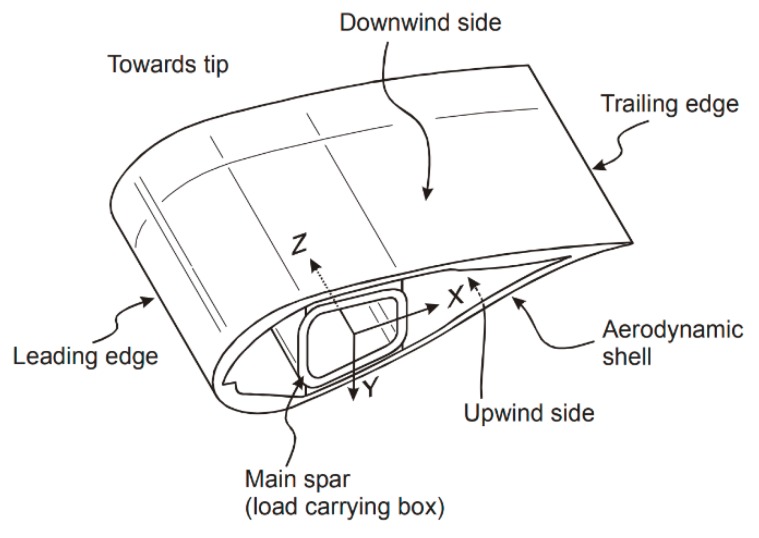
Blade of the wind turbine [37].

Despite the possible diverse wind turbine configurations, the horizontal-axis wind turbine (HAWT) with three blades is the most common design. The blades mainly consist of two shells that are bonded with adhesive and made of composite material [6,34]. The two shells form an airfoil shape that has a leading and a trailing edge as shown in Figure 2.

Usually, the shells are made of thermosetting polymer matrix composite, either polyester or epoxy, reinforced mainly with fibreglass. Carbon fibre is often incorporated in the main structural element to improve the blade deflection resistance [38]. The composite design, i.e., thickness and layout of laminates, can be varied across the blade to meet the requirements of certain blade areas [38,39]. Also, large wind turbines often consist of thickening sandwich materials such as wood or polymer foams, which are mainly used to add thickness to the blade and prevent buckling [6,40].

Although the use of composites as blade materials resulted in greater aerodynamic efficiency, they perform poorly under transverse and impact loads. They are also sensitive to environmental factors such as moisture, heat, erosion among others [38]. Moreover, there is a growing interest to use thermoplastic matrices instead of thermosets [41]. In theory, although thermoplastics provide better impact and erosive resistance compared to thermosets, the interest in the use of thermoplastic is mainly centred on the potential of recycling more than mechanical performance [41]. There is also an emerging trend in using advanced material such as composites reinforced with nanomaterials [42]. This is believed to improve the strength and impact resistance of blades.

### 3.2. Leading Edge Erosion (LEE)

**Figure 3:**
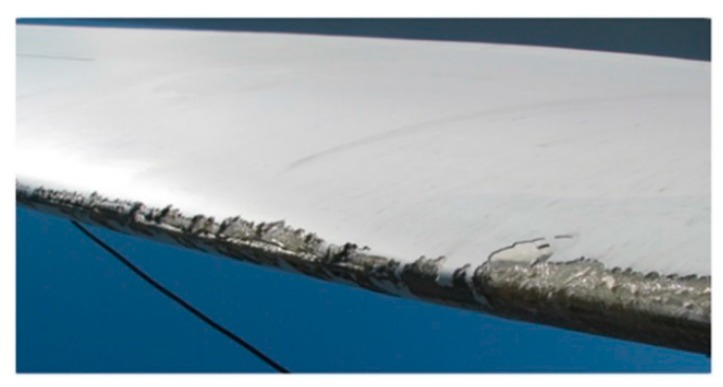
Leading edge erosion [43].

**Figure 4:**
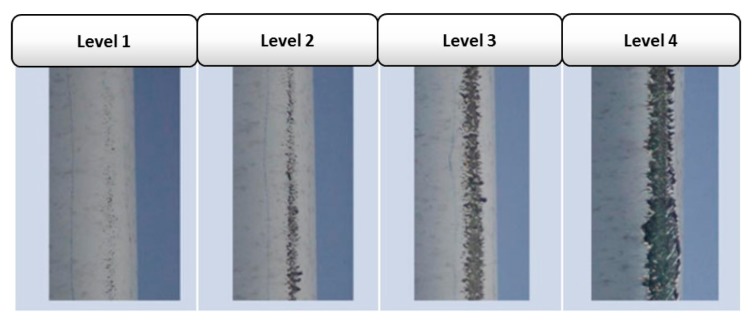
Level of erosion damage experienced at different radii. Adapted from [44].

Although erosion damage is sometimes seen in different places in the wind turbine blade, it is particularly significant at the leading edge of the blade [43]. Figure 3 shows an example of leading edge erosion of an actual wind turbine blade. Along the leading edge itself, the intensity of erosion damage varies depending on the distance from the rotor, as illustrated in Figure 4. This is mainly due to the linear speed gradient experienced at different radii. Eisenberg et al. [44] described the characteristics of the erosion damage shown in Figure 4 as follows:

- In level 1, only minor pitting introduced in the topcoat.
- In level 2, the underlying epoxy is intermittently visible. However, the topcoat has not been completely removed.
- In level 3 and level 4, the topcoat is completely removed and the epoxy is fully exposed with widths of damage less and greater than 15 mm, respectively.

It can be seen that the tip experiences the most severe erosion compared to other areas of the leading edge mainly because of the high linear speed (greater than 80 m/s) the tip can attain. Such material loss can be caused within just two years of service [6]. 

**Figure 5:**
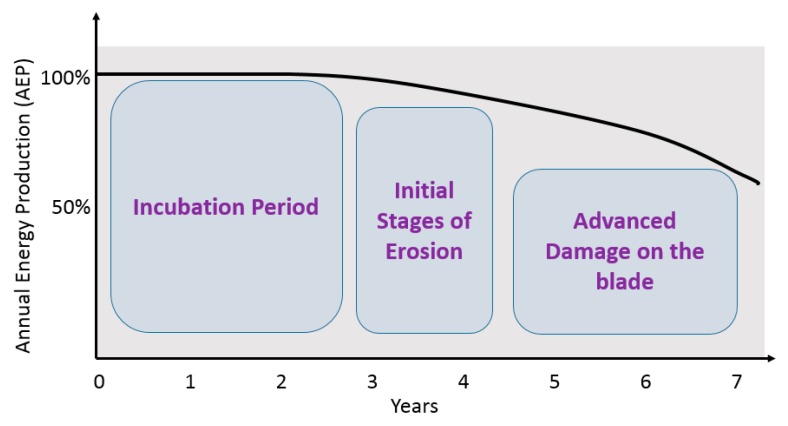
Annual energy production loss over turbine lifetime. Redrawn from [44].

The high surface roughness and deep pits caused by leading edge erosion result in a substantial increase in drag force coupled with a decrease in lift [45,46]. Hang et al. [47] showed that for a blade subjected to LEE, the drag and lift coefficients may increase and decrease by 314% and 53% respectively. Consequently, this can result in a remarkable reduction in the aerodynamic efficiency of the blade leading to lower annual energy production (AEP) [48]. Studies have shown that deeply eroded blades can result in as much as a 20% reduction in the rated power of the turbine [49,50]. Figure 5 shows the decrease in AEP as LEE problem progresses.

### 3.3. Erosion Conditions at the Leading Edge

#### 3.3.1. Impact Velocity 

**Figure 6:**
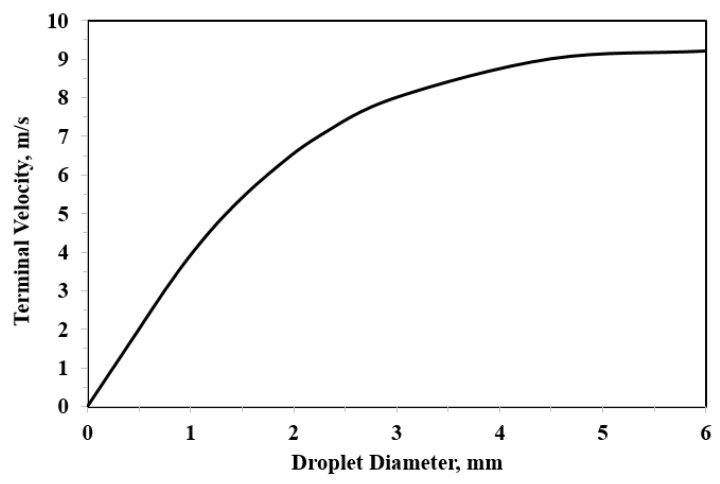
Dependence of terminal velocity of free-falling droplets on droplet size [6].

The impact velocity at the tip of leading edge depends on the peripheral speed of the tip and the terminal velocity of the incoming rain drops. The velocity of the falling droplet is dictated by the drop mass (size and density) as well as climatic conditions (temperature, humidity, and wind). Gunn and Kinzer [51] measured the terminal velocity of free-falling droplets of varying sizes through a stagnant air as shown in Figure 6. Another attempt to model the terminal velocity of the free-falling rain drop through static air was made by Wood [52], where the following equation was proposed:

(1) $$v_{t}\;≅\;\sqrt{\frac{\rho }{\rho_{a}}\;g\;D_{0}\;},$$

where $v_{t}$ is the terminal velocity, $D_{0}$ is the stable raindrop diameter, g is the gravity, and ρ and $\rho_{a}$ are the densities of the raindrop and air, respectively.

The actual impact velocity is a combination between the blade rotational speed and the terminal velocity of the falling raindrop. Keegan et al. [6] performed a simple vector calculation to establish an approximate value of the impact velocity for given operating conditions. They [6] showed that, if a terminal droplet velocity of 8 m/s is assumed, the impact speed does not drop below a value of 80 m/s even when the blade is rotating in a downward direction. 

#### 3.3.2. Droplet Size

The diameter of the raindrops depends on the climatic conditions under which they are produced as well as the conditions of the air carrying the droplets [6]. This, in most of the cases, results in a nonhomogeneous raindrop size, which is better described by a continuous distribution that indicates the number of raindrops with a specific diameter in a unit volume of air [53]. In addition, the prevalence of a particular diameter is often correlated to the intensity of the rainfall. Best [53] provided an equation to obtain the probability distribution of rain droplet diameter for particular rainfall intensity.

Historically, many researchers who studied rain erosion problem often favoured the use of 2 mm as a standardized droplet diameter for erosion tests [8]. This is because typified testing conditions were needed to compare and correlate erosion results of different testing facilities [25]. The comparison, however, has not been successful due in part to the different aerodynamic environments prevailing in different testing facilities [54]. It was also thought that 2 mm droplet diameter is suitable for testing since it corresponds to rain intensity of 25.4 mm/h. Rain intensity of 25.4 mm/h is classified as heavy rain [55], and it was believed that the majority of erosion damages occur during heavy/extreme rain conditions. By selecting 2 mm droplet size, a compromise is made between the greater individual effects of large drops and the greater number of impacts of the small drops [56]. This has been a reasonable assumption for experimental erosion testing practices. 

#### 3.3.3. Droplet Shape

In most of the cases, a raindrop is considered to have a perfect sphere shape. This is usually the case for droplets with a diameter of less than 2 mm. Villermaux and Benjamin [57] have shown that the shape of the raindrop depends on its diameter. For a diameter up to 2 mm, the raindrop remains spherical. However, between 2 mm and 5 mm, the shape is semi-oblate (flat at the bottom portion of the drop). For more than 5 mm, a drop is likely to have a parachute-like shape. Falling raindrops of more than 10 mm diameter usually disintegrate and fragment into smaller droplets [57]. Moreover, the stability of the falling raindrop is usually dictated by its velocity and the airflow condition. 

The frequency and intensity of on-site rain droplets impact loading on the turbine blade are difficult to define. Some researchers [58] attempted to develop a rain texture model that allows the transformation of statistical data of rainfall history at specific locations into 3D fields of raindrops. Such studies are needed to comprehend the nature and frequency of impact loading occurring on-site so that more representative laboratory experiments can be designed. This is also important in developing correlations between experimental results representing real in-service erosion experiences.

Knowing the impact conditions is important to study the interaction between water droplets and the turbine blade. This is because the physics of the impact and the damage created depend primarily on these conditions, i.e., impact speed, droplet diameter, etc. The following section outlines the physics of a single droplet impact as well as the mechanisms of erosion damage.

## 4. Physics of Droplet Impact

### 4.1. Impact Event

The physics of the water droplet impact erosion phenomenon is complex. The behaviour of a single droplet is often studied to understand the liquid-solid impact interaction. The following discussion summarizes the sequence of events taking place during the impact of a single droplet on a solid target.

#### 4.1.1. The Impact Moment

It is believed that when a liquid droplet impacts the solid surface, pressure pulses are generated inside the liquid and close to the liquid/solid interface [59]. These pressure pulses are responsible for the formation of a shocked envelop (disturbed region) in the liquid that only lasts for a few microseconds before breaking away. This is usually referred to as impact or “stagnation” moment. Two important phenomena occur during the impact moment; water hammer pressure and subsequent propagation of stress waves.

#### 4.1.2. Water Hammer Pressure

Cook [60] postulated that constant pressure is induced on a solid surface when it is being impacted by liquid jet or droplet. He [60] named this pressure as “water hammer pressure”. Cook proposed the following equation to calculate the water hammer pressure:

(2) $$P=\;\rho_{0}\;C_{0}\;V,$$

where $\rho_{0},\;\;C_{0},\;\;\mathrm{and}\;V$ are the liquid density, the speed of sound, and the impact speed of the droplet respectively. Following Cook’s water hammer equation, impact pressure has gained considerable attention because it represents the primary “loading” caused by droplet impact. The equation proposed by Cook [60] represents a uniform one-dimensional pressure and ignores the influence of the shock wave velocity variable for rigid and elastic surfaces. Dear and Field [61] attempted to account for the effect of target shock behavior on the magnitude of the impact pressure by the following equation:

(3) $$P=\;\frac{\rho_{0}\;C_{0}\;\rho_{s}\;C_{s}\;V}{\rho_{0}\;C_{0}+\;\rho_{s}\;C_{s}},$$

where the subscript s refers to the solid body variables. It is then concluded in several studies [62,63,64,65] that the impact pressure has spatial and temporal distribution in the impact zone, as well as a maximum peak value that occurs at a critical contact radius ($r_{c}$). The maximum peak value was seen to be more important than the full pressure distribution and many equations to quantify the maximum peak pressure were provided, such as Heymann’s approximation [63]:

(4) $$P=\;3\;\rho_{0}\;C_{0}\;V.$$

The full temporal and spatial distribution of the impact pressure was provided by many numerical works [65,66,67,68,69], where some such as Rosenblatt et al. [65] confirmed Heymann’s approximation of the maximum peak pressure. Once the impact pressure is obtained, it is usually used as a boundary loading condition acting on the solid target.

#### 4.1.3. Stress Waves

**Figure 7:**
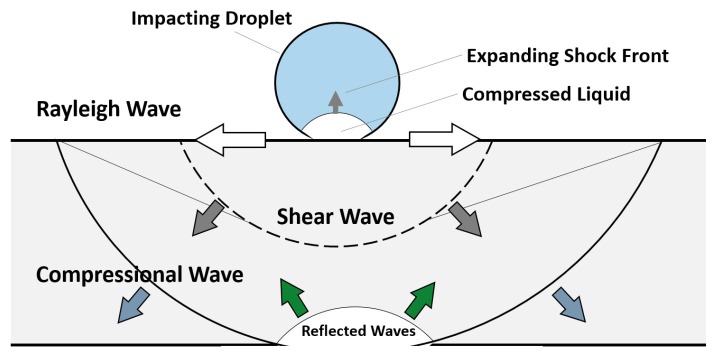
Schematic of shock wave behavior during the liquid droplet-solid surface interaction. Redrawn from [25].

The mechanical equilibrium (i.e., state of stress) in the target material is disturbed by the droplet impact and the pressure it creates on the impact zone. Three stress waves emerge from the impact zone to propagate this disturbance to the rest of the solid target, and therefore, shape its stress and strain field. These waves are a dilatational wave travelling in a longitudinal direction, a shear wave travelling in a transverse direction, and a Rayleigh wave moving along the surface [59,70]. Figure 7 illustrates the directions and the interaction of these stress waves. Velocities of these waves depend on the properties of the target, mainly elastic modulus, Poisson’s ratio, and density. The fracture can take place in the solid target due to passage of stress waves having high amplitudes of sufficient duration in excess of the dynamic fracture strength of the target. Stress waves can also interact with microstructural discontinuities resulting in the formation of high tensile stress due to stress concentration [71]. Therefore, stress wave propagation is considered as one of the main mechanisms with which high-speed droplet impact can cause failure.

#### 4.1.4. Lateral Jetting

**Figure 8:**
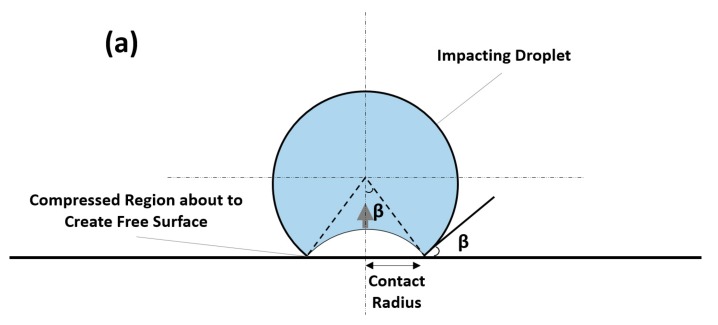

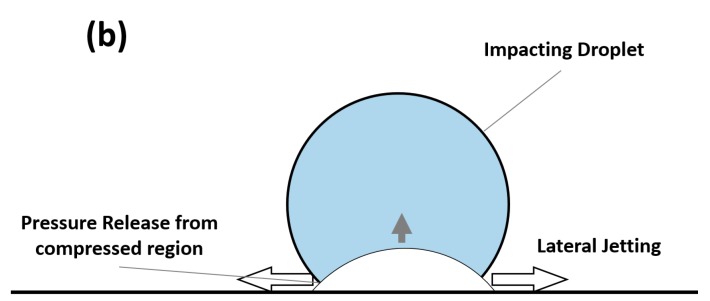
Schematic of the beginning of the lateral jetting formation.

**Figure 9:**
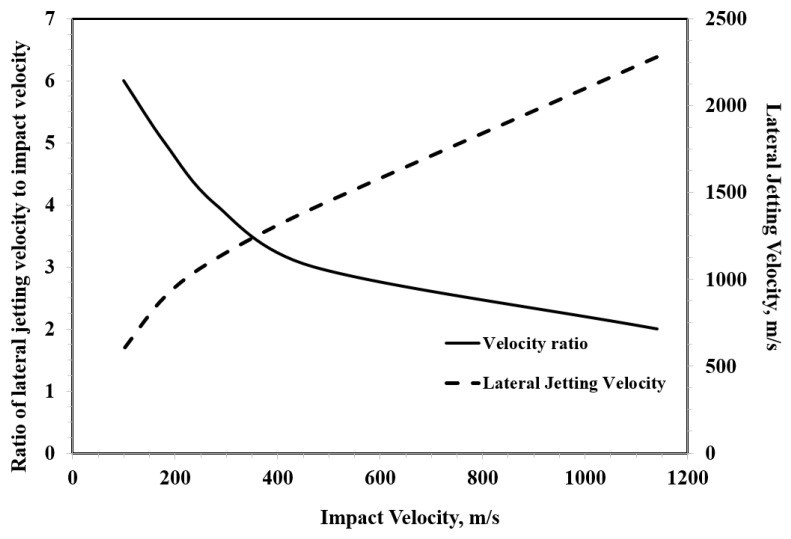
Lateral jetting velocity as a function of impact velocity. Redrawn from [76].

As a first step of depressurizing the droplet, lateral outflow or jetting erupts from the contact zone shortly after impact moment. The jetting is expected to start when the shock front of the compressed liquid region propagating inside droplet creates a free surface, as illustrated in Figure 8. This is expected to begin when the contact line velocity becomes equal to that of the propagating shock front [72]. It is reported that the velocity of the lateral jetting can reach up to several times that of the impact speed [59,69,73]. Jenkins and Booker [74] measured the velocity of lateral jetting for a 2 mm water droplet over a range of impact velocity of 100 to 1140 m/s. Figure 9 shows the ratio of lateral jetting velocity to impact velocity and the corresponding lateral jetting velocity based on Jenkins and Booker’s [74] experimental results, where the high ratio can be noticed especially at impact speed less than 400 m/s. It can also be noticed from Figure 9 that the value of the lateral jetting velocity increases with the increasing the impact velocity. From a tribological point of view, and because of this high velocity, the lateral jetting plays an important role in the initiation of the erosion damage as it can potentially tear surface irregularities. According to Najafabadi et al. [75], surface asperities greater than 100 nm are likely to be broken by the liquid jet. This is why the surface quality and roughness play a very important role in the initiation of erosion damage.

**Figure 10:**
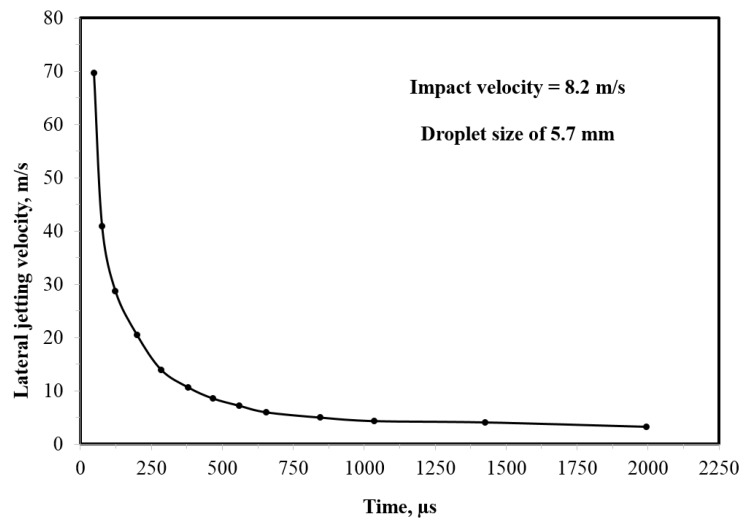
Time dependency of lateral jetting velocity at specific impact event. Redrawn from experimental data of [77].

It is also worth mentioning that lateral jetting velocity is time-dependent. Experimental measurements of Jenkins and Booker [74] (Figure 9) would only measure the average velocity of lateral jetting at specific impact velocity. The time dependence of lateral jetting velocity was clearly demonstrated in the work of Engel [77], where lateral jets of free-falling droplets were observed by a high-speed camera. The emerging lateral jet begins with a very high velocity that decreases with time to eventually reach a value below that of the impact velocity, as shown in Figure 10. The experiment shown in Figure 10 was performed at relatively low impact velocity (less than 10 m/s), and it is still not known if similar behaviour holds at higher impact velocities. The shearing action that can be caused by the significantly high lateral jetting velocity at the beginning of jet formation could potentially be viewed as the principal cause of the ring fracture seen in the failure of brittle materials due to single impact of a high speed water droplet (see for example [78]). Therefore, the lateral jetting velocity at the beginning of jetting is significant. Engel [77] provided an equation according to which the lateral jetting $v_{l}$ immediately at the beginning of jetting can be estimated from the impact velocity V, as follows:

(5) $$v_{l}=\;\sqrt{\alpha \;c\;V},$$

where c is the speed of sound in the impacting liquid and α is a coefficient that determines the fraction of the impact velocity imparted to the liquid molecules. This will mainly be determined by how much of the liquid the compressional wave occupies as it spreads through the spherical drop and creates the free surface for the jetting to emerge [77]. The value of α approaches unity as the velocity increases. Even when a value of unity is chosen for the coefficient α, Equation (5) suggests that lateral jetting velocity would be less than the impact velocity as it approaches the speed of sound in the liquid, which is contrary to what is observed in Figure 9. Evidently, understanding the behaviour of lateral jetting is of crucial importance to understanding the water droplet erosion mechanisms, which is still lacking.

The damage progression and the physics of droplet erosion become even complex when the repetitive impact nature of the problem is considered. Until now, a convincing erosion theory that takes into consideration all these aspects has not been developed. The following sections outline the time dependency of erosion damage, some of the interpretations regarding erosion mechanisms, and the influence of impact parameters on erosion behaviour.

### 4.2. Time Dependency and Stages of WDE Damage

**Figure 11:**
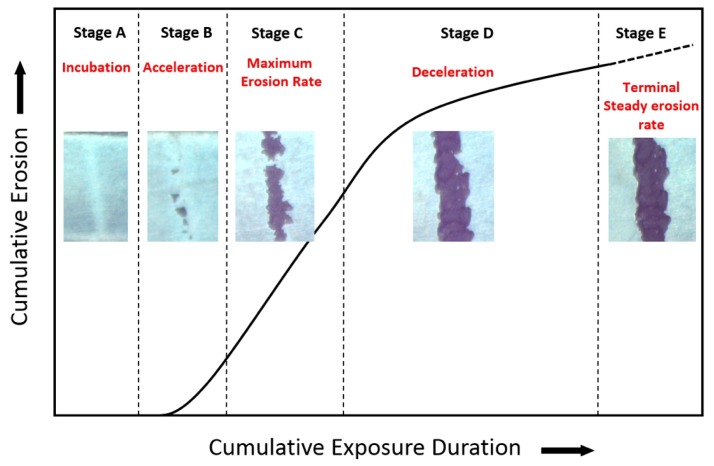
Typical stages in water droplet erosion damage curves. The insets are Ti64 tested at 300 m/s impact velocity with droplets of 460 µm size.

A long history of experimentation has shown that water droplet erosion is a time-dependent phenomenon, i.e., it exhibits different erosion rates at different time intervals, resulting in a nonlinear progression of damage [79]. This is usually presented in the so-called erosion graphs. Figure 11 shows a schematic of the typical (called s-shaped) erosion curve observed during the water droplet erosion of almost all bulk materials. ASTM G73-10 [80] and Heymann [14] divide the erosion curve into five distinct regions or stages. Actual images of each erosion stage experienced during the erosion process of Ti-6Al-4V alloy are shown in Figure 11 for representation. The first stage, stage A, is called the incubation period. In this period, there is only an increase in the roughness of the surface due to the repetitive impact of the water droplets without measurable material loss. The incubation period is sometimes very short or may not be seen in the case of extreme test conditions. Then, micro-pits start to emerge with more droplets impacting the surface, resulting in a measurable material loss. The material loss starts with a continuously increasing rate (stage B) in what is described as the acceleration stage. In this stage, the generated pits coalesce, which leads to an increase in the size of the crater. At the end of the acceleration stage, the erosion rate increases to its maximum, and remain constant for a relatively long period (stage C). This stage of constant erosion rate is known as steady-state maximum erosion rate and it is very important in characterizing the erosion damage. After the steady-state erosion and once the erosion crater attains certain depth, the erosion rate starts to decrease (usually 25% to 50% of the maximum erosion rate [14]) in the so-called deceleration or attenuation stage (stage D). This decline in the erosion rate is attributed mainly to the high roughness and irregularities encountered in the erosion crater, which effectively changes the stress distribution, and therefore reduce the erosion rate. The deceleration of the erosion rate continues until it once again becomes constant in the terminal stage (stage E). Some tests do not show terminal stages, and erosion rate either continues to decline or exhibits a series of fluctuations [14]. In some brittle materials and coatings, the erosion rate after the deceleration period starts to rapidly increase in what is called the catastrophic stage [14]. 

There has been a discussion around which erosion stage is the most important from a practical viewpoint. On the one hand, it was argued that since components spend most of their lives in deceleration stage (stage D), the erosion rate at that stage should be used as a measure of erosion damage [81]. On the other hand, others [82,83] argued that the level of erosion damage at stage D can be so serious that components not only lose their primary function but can no longer be serviceable. Therefore, the erosion rate at stage C and/or the length of the incubation period should be used to characterize the erosion damage. This is why most of erosion prediction models attempt to quantify incubation stage and the constant erosion rate at stage C. It is also to be noted that the reason for this pattern in erosion graphs is still not clear. It is likely that physical nature of the damage and the change in the geometrical surface (i.e., the shape of the evolving crater) taking place during erosion progression are responsible for the pattern seen in erosion graphs, however, this still needs to be studied.

### 4.3. Erosion Damage Mechanisms

Extensive research has been carried out to understand the mechanisms responsible for the initiation and progression of erosion damage due to single or multiple droplet impacts. Generalized conclusions have not yet been made. What is known so far is that erosion seems to have damage regimes. That is, the erosion damage mechanism that is operative depends largely on the impact conditions (especially impact velocity) and the dynamic response of the target material [76]. The initiation of erosion is often seen as an important issue and range of explanations [70,84,85] has been given to describe the sequence of events that lead to the beginning of weight loss in the material. For polymeric materials, which are the primary concern in the wind turbine leading-edge erosion LEE problem, erosion initiation and progression can be explained as a result of one or more of the following mechanisms [76,86]:

- Direct deformation at high speed impacts.
- Stress wave propagation.
- Surface fatigue due to the repeated loading caused by droplet impacts.
- Initial pits and dents are caused by former impacts and erosion is then caused by lateral jetting and hydraulic penetration.
- During heavy rain, the high frequency of droplets impact does not allow the material to recover fully from one impact before the next impact event.

Surface fatigue received considerable attention as the damage mechanism [87,88] responsible for the initiation of erosion in wind turbine blades. This was mainly due to three factors; firstly, the repetitive nature of the loading caused by the impacting water droplets. Erosion damage is observed only after several impacts instead of a single impact, so long as the impact velocity is below the fracture threshold velocity. It follows that stress is being accumulated during an incubation period until cracks are initiated and thus erosion fractures are observed. The second indicator of fatigue similarity might have been the existence of a lower threshold speed (or more precisely, erosive conditions) below which erosion is not likely to take place. This was experimentally observed in several studies (for example [89,90,91,92]) and it resembles the idea of endurance limit in fatigue failure. Finally, microscopic observation of erosion damage often reveals the presence of fatigue marks. This may have led researchers [44,93,94] tackling leading-edge erosion of wind turbine to consider fatigue as the primary mechanism behind the erosion initiation. 

It is worth mentioning that, for a long time, fatigue was considered to be the dominating damage mechanism in the erosion of metallic materials. However, Adler [76,95], after a closer look at the microstructural damage caused by erosion, pointed out that fatigue plays only a secondary role in WDE damage. He suspected that initial impacts are responsible for topological changes in the surface while the damage is caused mainly by lateral jetting and hydraulic penetration. He also found, by examining the cross-section of erosion craters through progressive sectioning, that cracks front are quite blunt, indicating a tunnelling process rather than simple crack propagation. Moreover, other researchers [96,97,98] undermined the role of fatigue in erosion failure without completely dismissing its existence. This was concluded when various surface treatments, namely deep rolling, laser shock peening and ultrasonic nanocrystalline modification, known to remarkably improve fatigue life showed no or little improvement in erosion resistance. Thus, further confirming Adler’s conclusion. 

Although this was concluded mainly for metallic materials, LEE researchers may need to be careful in drawing inferences about the domination of fatigue in all erosion regimes of polymeric materials. That is, some materials and erosive conditions combination may lead to fatigue failure, but others may not. Alternative justification such as considering the ability of the material to recover before the subsequent impact event and the role of surface characteristics with respect to lateral jetting can be considered and further investigated for erosion behaviour of polymeric materials. This is because materials that can elastically recover quickly usually exhibit high erosion resistance [86]. Thus, more studies are needed to understand the role of damping and materials ability to recover as well as the role of lateral jetting on erosion behaviour. This may be achieved by a closer look at the interaction between the impacting water droplet and dynamic properties, and the changing topography of materials.

### 4.4. Effects of Erosion Parameters

Droplet impact erosion process is significantly influenced by impact parameters such as impact velocity and droplet size as well as target mechanical and surface properties. This because erosion parameters and target properties dictate the amount of water hammer pressure, stress waves amplitudes and speeds, and lateral jetting, which in turn control the nature and mechanisms of erosion damage.

Several studies [21,62,89] indicated that the impact velocity is the most influencing parameter on water droplet impact erosion. For example, experimental data revealed that erosion rate is exponentially proportional to impact speed according to one of the following relationships [99]:

(6) $$ER=a\;V^{n},$$

(7) $$ER=a\;{\left(V-\;V_{C}\right)}^{n},$$

(8) $$ER=a\;e^{nV},$$

where ER is the erosion rate, V is the impact velocity, a is a constant, n is the speed exponent, and $V_{C}$ is the threshold velocity below which erosion is not likely to take place. The value of speed exponent n in water droplet erosion can reach up to 10 [90]. For leading edge erosion applications, Eisenberg et al. [44] reported a speed exponent of n=6.7.

**Figure 12:**
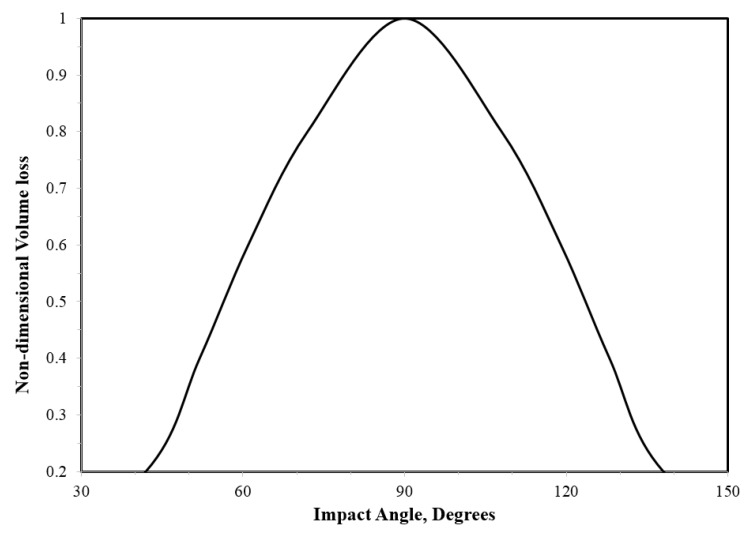
Non-dimensional volume loss of stainless steel as function of impact angle [33].

Impact angle is equally important and often related to its influence on the impact speed [14,33,100]. Ahmed et al. [33] demonstrated that 90° impact angle caused the maximum erosion damage for the same time of exposure, as shown in Figure 12. This is because 90° impact angle corresponds to the maximum normal component of the impact velocity. 

Droplet size was found to have a significant influence on WDE behaviour. It influences the distribution of the impact pressure, the area exposed to the impact (impact zone), the transferred kinetic energy, and time duration of each impact pulse [100,101]. According to the pressure-wave erosion theory, larger droplets are likely to cause more erosion than smaller ones for the same total volume of water [14]. Experimentally, several studies confirmed the effect of droplet size on the WDE, such as the work in [15,102,103,104]. For example, Hattori and Lin [102] concluded that the volume loss per droplet is proportional to droplet diameter raised to the power 4.7, which indicates the strong influence of the droplet diameter.

Surface properties of the target material are of equal importance. The initial surface condition was found to have a great influence on the initiation of the erosion. Several studies [29,78,99,105,106], demonstrated that the initial surface roughness can strongly influence the length of the incubation period. This is mainly due to the tearing of asperities and surface irregularities by the lateral jet emerging from the droplet. Hence, the smoother the surface the lesser shearing effect, and thus delayed erosion. Moreover, the existence of liquid film on the target surface has shown to have a cushioning effect on liquid impact erosion [107]. This because impact pressure as well as shear damages caused by lateral jetting are greatly reduced by the liquid film [107,108]. However, according to Shi et al. [109], with the progress of damage, the liquid trapped in pits or cracks can be forced to penetrate and cause higher damage than the dry surface. This discussion highlights the complexities brought about by the sheer number of parameters actively influencing erosion damage and the dynamic and transient nature of the problem. The next section discusses erosion experimentation techniques and data representation.

## 5. Experimental Testing of Water Droplet Erosion

Erosion tests are accelerated experiments where severe erosive conditions are artificially generated to produce rapid erosion damage. It is to be mentioned that erosion testing has not evolved to a stage where the quantitative prediction of the erosion performance of materials in actual in-service conditions can be made. This is mainly due to the lack of correlations between the testing environment and service life. Therefore, erosion tests are performed only to obtain a qualitative assessment of erosion resistance of materials as well as to understand their erosion behaviour. The following discussion highlights some of the important erosion testing facilities pertinent to the leading-edge problem, a brief discussion about recommended practice in testing for wind turbine blade protection systems, and erosion testing results and data representation.

### 5.1. Testing Facilities

**Figure 13:**
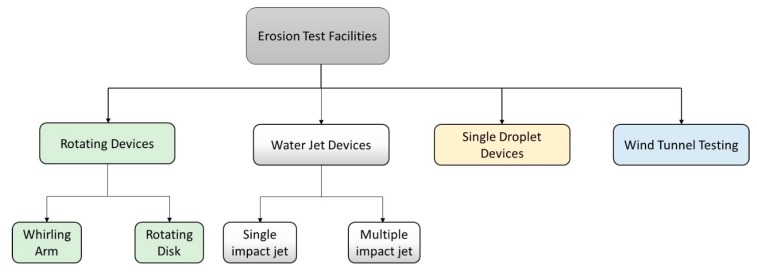
Classification of erosion testing facilities.

Many erosion testing facilities have existed throughout the literature. According to Hammit et al. [110], erosion testing facilities can be broadly classified based on two criteria: (i) whether the droplet is moving or stationary and (ii) either single or multiple impact devices [110]. There are essential requirements that should be satisfied by any erosion test facility. These include the ability to test different types of materials which are of interest in applications where rain erosion is a problem, i.e., elastomers, plastics, composites, metals and ceramics. Testing should also be simple, reliable and inexpensive, while realistically simulate rain erosion conditions in a controlled environment [110]. Based on these requirements, there have existed various rain erosion test facilities reported in the literature, notably rotating facilities [111,112], jet erosion facilities [113], single drop impact facilities [114], and wind tunnel erosion testing [115]. Figure 13 describes a general classification of water droplet impact erosion testing devices. Generally speaking, single droplet (or jet) impact devices help in establishing the physics and fundamentals of water droplet erosion phenomenon, whereas multiple droplet facilities (e.g., rotating rigs) provide a rather qualitative data about the erosion performance of materials. Gohardani [25] presented a compilation of rain erosion test facilities and thoroughly discussed their advantages and shortcomings. Rotating facilities are further discussed because they are the primary devices currently used to investigate leading-edge erosion (LEE) problem in wind turbines.

**Figure 14:**
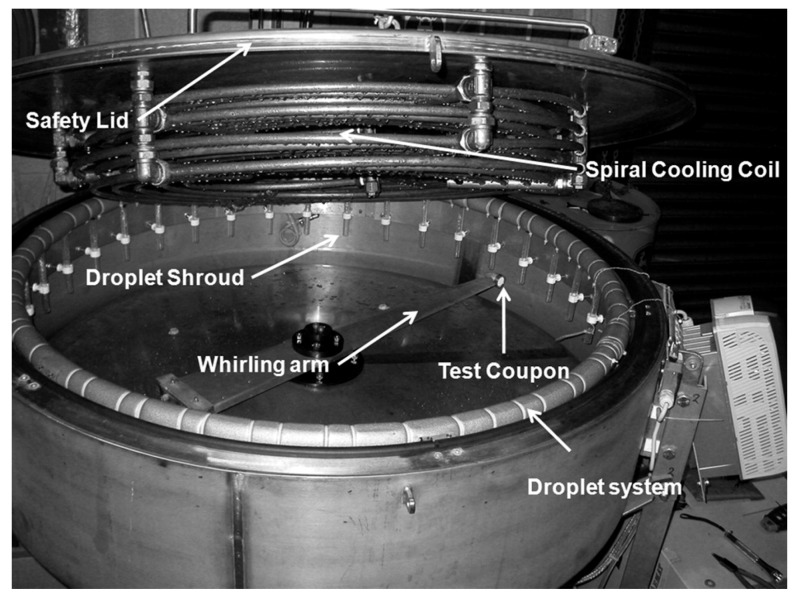
Example of Whirling Arm apparatus [117].

**Figure 15:**
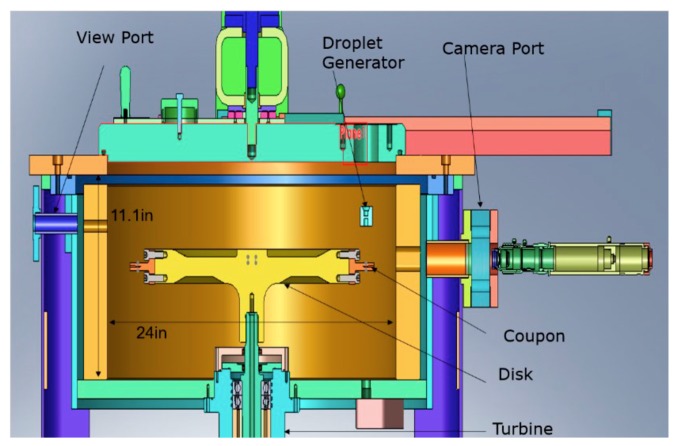
Example of Rotating Disc Erosion Test Rig.

Rotating devices can be classified based on the main rotating component into either whirling arm [116,117,118,119] or rotating disc devices [21,101,120]. In both cases, samples are mounted on the periphery of the arm or the disc, and nozzles are located immediately above the sample. These nozzles inject water droplets in such a way that droplets impact the sample with an angle of 90° in most of the cases. The impact speed is considered as the linear speed of the rotating component at the point of impact. The difference is that whirling arm facilities usually consist of a rotating propeller blade or arm, whereas rotating disk facilities usually consist of a well-balanced heavy disc, as shown in Figure 14 and Figure 15, respectively. 

Whirling arm devices have an acceptable level of reproducibility and allow for comparison of relative erosion resistance of materials. However, they are expensive, have a complex environment and in some cases allow only fixed mass and geometry of sample due to high level of vibrations. They also exhibit a high centrifugal force on the specimen. This is why whirling arm facilities usually have limitation in rotational speed [25], reaching an average maximum of about 150 m/s. On the other hand, rotating disk facilities can reach up to 500 m/s in some cases [21] or more due to the balanced rotating disc. The rotating disk is usually contained within a vacuum chamber that serves to minimize the temperature rise due to the friction between the rotating disc and air. The vacuum also helps in minimizing droplet distortion due to the turbulent circulation of air that is present in whirling arm facilities. Moreover, one nozzle is often used with rotating disk rigs, which helps in quantifying the exact number of droplets impacting the sample at each revolution [121].

As noted by Bartolomé and Teuwen [34], rotating devices are inherently incapable of replicating the movement of the turbine blade during rainfall impacts. The interaction between the raindrop and the wind turbine blades principally depends on the position of the blade during rotation. The blade position during the raindrop impact dictates the impact velocity [6], resulting in what might be multiple impacts of variable velocities. It may also influence the impact angle. The same level of complexity is present in WDE of gas and steam turbines but at higher impact velocities. Hence, simulation of the impact configurations in the wind turbine blade seems to be an issue for all water droplet erosion testing devices. Nonetheless, qualitative studies of erosion resistance and erosion behaviour of materials can still be conducted.

### 5.2. Recommended Practices for LEE Testing 

Different researchers in the past performed erosion testing using different approaches due to the absence of testing standards—that is, different testing variables are controlled and reported for different erosion testing facilities. This had prevented a useful comparison between the results obtained from different erosion facilities. The development of the first erosion standard (ASTM G73 standard [80]) was completed in 1982. ASTM G73 [80] provide detailed standard practices for liquid impingement erosion testing and has been followed by many researchers in the field including the testing of wind turbine blade protection materials. Recently, Det Norske Veritas (Norway) and Germanischer Lloyd (Germany) DVN GL society has developed a recommended practice (RP) guide particularly for rain erosion testing of wind turbine blade protection systems under the name of DNVGL-RP-0171 [122]. DNVGL-RP-0171 recommended practice was developed as an extension to ASTM G73.

Like ASTM G73 [80], DNVGL-RP-0171 [122] provides guidance as to which parameters will influence the test results, and therefore shall be monitored and controlled during erosion testing. DNVGL-RP-0171 [122], however, defines these parameters so as to represent the environmental condition that a leading edge of a wind turbine blade is exposed to. It [122] also provides guidance for the geometry of specimens to be tested as well as the preparation technique to be used. Moreover, it [122] provides the conditions of failure of a coating protection system as well as for protection tapes. This is very important because what is considered a failure (and necessitate a maintenance operation) differs for different erosion applications, and a specific definition is needed for the LEE problem.

Nevertheless, DNVGL-RP-0171 [122] ignores a detailed description of erosion failure beyond the end of incubation period. This can still be an important aspect in LEE problem especially when coating systems are considered. Overall, it [122] serves as an important guidance for rain erosion testing of WTBs to which further improvements can be made.

### 5.3. Data Representation

Erosion results are usually represented in terms of cumulative material loss versus the cumulative erosion exposure duration, as shown in Figure 11. The cumulative material loss (vertical axis) is either plotted using mass loss [98,123], or volume loss [124] so that different types of information can be captured. ASTM standard G73-10 [80] recommends the use of volume loss where the density of the material is used to convert the mass loss into volume loss. Mean depth of erosion (MDE) is also sometimes used to represent the cumulative material loss. Difficulties, however, arise with finding an adequate parameter to express the cumulative exposure duration (horizontal axis). ASTM standard G73-10 [80] recommends the use of the volume of water impacting the surface at a certain area. The use of volumes for both material loss (vertical axis) and exposure duration (horizontal axis) would result in dimensionless slopes in the erosion graph, which is sometimes useful when comparative studies are to be conducted. However, it is difficult to obtain the exact volume of water impacting the surface and previous studies utilized different parameters to represent the cumulative exposure duration. For example, erosion time has been the most commonly used parameter [33,98,125]. Other parameters used were the number of revolutions in rotating devices [126,127] and the number of droplets impinging the surface [21,96]. The problem with using these parameters is that the influence of droplet size, impact speed, frequency of impact, etc. on the erosion results is overlooked. Thus, erosion time, number of revolutions, and (to a lesser degree) number of impinging droplets are not reliable parameters for reporting erosion data. 

Kirols et al. [121] proposed the use of impact energy intensity as the main parameter to represent cumulative exposure duration (horizontal axis) of erosion graphs. The impact energy intensity was considered as the kinetic energy of the impacting droplet normalized to the impact area measured by microscopic observation. The kinetic energy of the impact is calculated simply using the following equations:

(9) $$K_{E\;}=\;\frac{1}{2}\;m\;v^{2},$$

where,

(10) $$m=\;N_{droplet}\;\rho_{water\;}\;V_{droplet}$$

v is the impact speed,$\;\rho_{water\;}$ is the density of water, $N_{droplet}$ is the number of droplets impacting the surface, and $V_{droplet}$ is the volume of one droplet (the droplet is assumed to be spherical). This way of calculating the impact energy intensity allows the consideration of impact conditions and droplet characteristics. The assumption was that, theoretically, the same applied energy should cause the same level of erosion, regardless of the individual parameters used in the experiment. The success of using impact energy intensity was limited because it was believed that a portion of the total kinetic energy is dissipated upon the impact. The amount of energy dissipated, they argue [121], is a function of the erosion conditions (such as impact speed, droplet size, etc.) as well as the morphology of the eroded surface, i.e., the shape of the evolving erosion crater. They proposed a severity coefficient (ξ) that serves to quantify the energy dissipation behavior. However, the physical meaning of the severity coefficient and how it can be calculated from the erosion conditions and target properties were not studied. 

One of the drawbacks of the energy intensity approach is that it ignores the temporal and spatial statistics of the impact. That is, it ignores the frequency of impact per specific site and the distribution of the impact intensities over a certain area. This is particularly important in studying the erosion behaviour of elastomeric material, where the material is required to recover from one impact before the next droplet impact. Hence, the frequency of impact becomes an important parameter in erosion damage. 

It can be concluded from the above discussion that erosion data representation remains a challenging issue. Most of the parameters used to represent the cumulative exposure (horizontal axis) have limitations in considering the parameter of the erosive environment. Nonetheless, the idea of applied energy intensity can form a good basis towards further developing a universal method of data representation, if the impact statistics are properly incorporated.

## 6. Modeling and Prediction of Erosion

### 6.1. Erosion Model Formulation and Challenges

**Figure 16:**
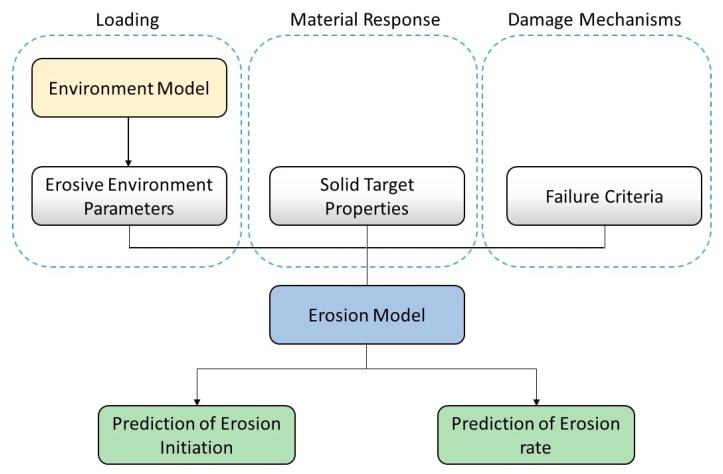
Formulation of a simple erosion prediction model.

Generally, modelling water droplet erosion involves the development of mathematical models that can predict erosion initiation and rate of progression. This breaks down mainly into three tasks; characterization and evaluation of the intensity of the erosive environment in terms of impact loading, definition and measurement of the relevant target properties and its response to impact, and adoption of representative failure criteria. Figure 16 shows a general schematic of the formulation of the erosion prediction model. 

The erosive environment can be represented in terms of a model where parameters such as impact speed, droplet size, frequency of impact, etc. can be the input. This may represent a metrological data related to rainfall rates and droplet size distribution or may represent the erosive environment of a certain test facility [76]. Then the characteristics of the impact pressure caused by droplet impact and the consequent temporal and spatial stress and strain fields in the solid are obtained. This has received considerable attention in the period between the 1960s and 1980s and many analytical and numerical models were developed, notably Blowers’ analytical solution [64], Hwang [128], Rosenblatt [65,129], Adler [114], and Lesser [130]. A comprehensive review of the analytical and numerical efforts done to characterize the impact pressure distribution and stresses in the solid was reported by Lesser and Field [131]. The main difficulty facing the characterization of the loading and the stress field has been the coupled liquid-solid interaction encountered in water droplet erosion problem. 

Equally disconcerting, has been the proper selection of target properties pertaining to materials’ resistance to water droplet impact erosion (i.e., erosion strength). Attempts were made to use different material properties such as strength (yield or ultimate), hardness, toughness, hardenability, and ultimate resilience to represent erosion resistance [99,103,132,133]. The challenges have been to, firstly, define the proper number of properties and their adequate combinations, and secondly whether a set of properties applicable to a certain erosion intensity would still be valid at widely different intensities where microscopic failure modes are likely to change. Surface topological parameters also seem to be very important to water droplet erosion phenomenon as experimental evidences have shown [29,105,114]. Yet, surface features have not received greater attention when modelling is concerned.

Finally, the question of damage mechanisms remains as one of the most enduring puzzles of water droplet erosion problem. A comparison between impact stresses propagated by waves and the dynamic fracture strength of the target material was often used to explain the mechanism behind crack initiation due to single droplet impact. Complexities, however, appear when mechanisms of cumulative damage due to multiple impacts are considered. As mentioned earlier, many of the researchers [44,89,93,134] working on rain erosion were inclined towards surface fatigue as the main damage mechanism. It was suggested [87,88,135] that if the obtained information about the stress field due to an impact event is superimposed with the impact statistics (number of impacts), a fatigue analysis can be performed to predict erosion damage caused by water droplet impacts. This is why the two erosion prediction models developed to a useful state of completion were Thiruvengadam’s model [87,136] and Springer’s model [88,137], which are based on the concepts of fatigue life of materials. These two models, although have been developed to correlate a broad erosion database, were contested in the literature as they failed to predict erosion in many other applications [76]. A brief discussion about Springer’s models and how it is used in the context of leading-edge erosion LEE of wind turbine blade is presented in the next section.

### 6.2. Fatigue Models for Leading Edge Erosion

An earlier attempt to develop a generalized rain erosion damage model based on fatigue similarity was made by Springer [88,137,138,139]. Researchers [103,140] before Springer also attempted to use fatigue similarities to develop erosion models, however, their models remained applicable to limited experimental data. Springer’s models, on the other hand, were proven to correlate a considerable amount of erosion data, especially at subsonic impact speeds. The model was first developed for homogenous materials [88], and it was then extended for composite materials [138], coatings [137], and electromagnetic transmission losses in transparent materials [139]. 

**Figure 17:**
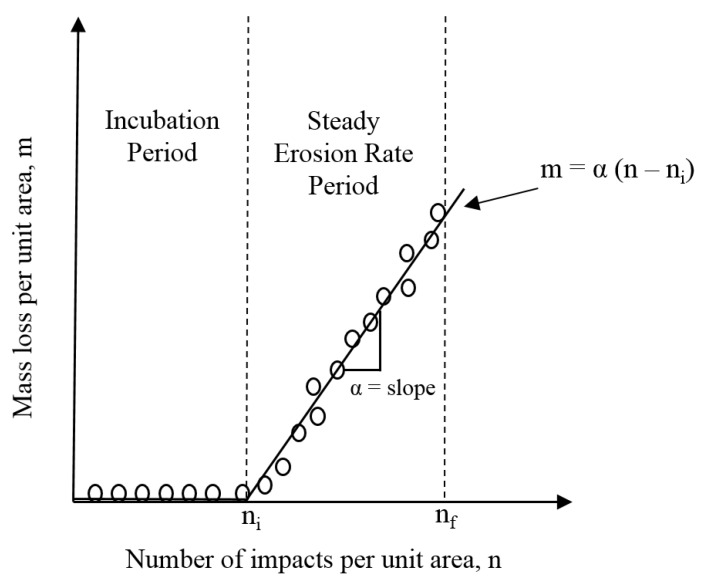
Schematic of Springer’s solution model. Redrawn from [134].

Springer [134] postulates that the erosion curve of materials, i.e., mass loss (m) vs number of impacting droplets (n), can be represented by two straight lines as shown in Figure 17. According to this approximation, the mass loss can be given by:

(11) $$m=0\;\;\;\;\;\;\;\;\;\;\;\;\;\;\;\;\;\;\;\;\;\;\;\;\;\;\;\;n<n_{i},$$

(12) $$m=\;\alpha \;\left(n-n_{i}\right)\;\;\;\;\;\;\;\;\;\;n_{i}<n<n_{f},$$

where the subscripts i and f represent the end of incubation period and the end of maximum erosion rate stages, respectively. Therefore, the problem reduces to finding $n_{i}$, α, and $n_{f}$ from the impact conditions and proper materials properties, and consequently the mass loss (m). The analysis used to find these parameters is based on the linear cumulative fatigue damage rule of Palmgren–Miner [141]. Derivation and final equations can be found in Springer’s book [134]. It is important to mention that Springer model neglects the effect of some important materials properties such as hardness and surface quality. It also neglects the contribution of lateral jetting to the erosion process, which has been proven to immensely influence the erosion process. Despite all these limitations, Springer model seem to correlate well with polymeric materials and coatings at subsonic impact speeds. This is why it is being adopted for predicting the erosion loss in the leading edge of wind turbine blades. For example, Eisenberg and co-workers [44] at Siemens Gamesa recently applied Springer’s [134] erosion model of coatings to predict erosion initiation in both testing environment and field conditions. This was feasible mainly due to their access to real-life erosion observations as well as historical rain fall data at specific site and turbine rotations rates. According to them [44], the model successfully correlated with the erosion data of the coating they examined. 

A recent attempt to model the life of leading-edge coatings that are subjected to rain erosion was made by Slot et al. [93]. Like Springer’s, this model is based on the linear cumulative damage rule of Palmgren–Miner [141]. The primary source of loading in this work was considered to be the stresses due to the Rayleigh surface wave generated by droplet impact. The model is developed only to predict the end of incubation period ($I_{p}$), which is given by the following equation:

(13) $$I_{p}=\;\frac{D_{f}}{D_{h}}$$

where $D_{h}\;$ and $D_{f}$ are cumulative fatigue damage per hour and cumulative fatigue damage at failure respectively. $D_{h}$ depends on the maximum stress due to Rayleigh surface wave ($S_{max})$ and fatigue limit at standardized test conditions ($S_{D})$ and is given by:

(14) $$D_{h}=\;\frac{12\;I_{r}}{d_{d}}\;\frac{A^{2}}{{\left(h_{tot}\;S_{f}\right)}^{m}}\;\frac{1}{\left(m-1\right)}\;\left[S_{max}^{\left(m-1\right)}-\;h_{tot}S_{D}^{\left(m-1\right)}\right],$$

where $d_{d}$ is the droplet diameter, A is the impact area, $h_{tot}$ is a factor that corrects for the difference between standardized fatigue test conditions and actual rain impact conditions, m and $S_{f}$ are material parameters commonly used for studying fatigue. It is to be mentioned that $h_{tot}$ is an adjustable parameter that has no constraints or physical meaning. Also, Slot’s et al. [93] model ignores erosion damages caused by compressional and shear waves and only considers the contribution of Rayleigh surface wave. Compressional and shear wave are particularly important when interfaces (coating systems) and structural inclusion are involved. Moreover, the model does not take into consideration erosion damage caused by lateral jetting.

### 6.3. Numerical Modelling of Leading Edge Erosion 

Similar to analytical and semi-empirical models, computational studies attempted to address droplet impact erosion by first modelling of the intensity of the erosive environment, i.e., impact parameters and the impact pressure it creates as a function of time and space, such as the work in [49,66,67,142]. Secondly, obtaining the stress and strain field generated in the solid target due to the impact pressure loading. Finally, the ensuing erosion damage is obtained by applying fatigue damage models in case of multiple impacts, such as in the work of [94,143]. Some researchers combined both types of analysis such as Amirzadeh et al. [58,94] and Zhou et al. [143,144].

Generally, three numerical modelling methods are employed to model short-duration high-speed impact [145,146]: (i) standard finite element techniques using Lagrangian meshing method. This method is widely used, however, it is less accurate when large deformations are expected; (ii) Eulerian/Lagrangian method, where the solid surface is modeled using Lagrangian method, whereas the impacting droplet is modelled with Eulerian method; (iii) Smoothed particle hydrodynamic (SPH), which is a meshfree Lagrangian method. In SPH, instead of meshing the cell, the material medium is represented by numerous small particles. These particles carry the properties of the fluid and move at its velocity, i.e., the coordinates move with the fluid. Also, these particles are not connected together directly, rather, an interpolatory scheme based on Kernel function [147] is used. This primarily results in short computational time. In addition, Cortés et al. [148] proposed the incorporation of cohesive zone modelling (CZM) for coatings and multilayered systems to consider the role of interfaces in the impact of erosion failure.

The use of numerical techniques to predict water droplet erosion damages is faced by many challenges regarding the effectiveness and generalization of these techniques. This is because physics and damage mechanisms of the erosion phenomenon have not been fully understood, and thus, an agreed upon analytical solution of WDE problem still does not exist. This renders most of the numerical work meaningless or only valid for specific data sets. However, as far as the leading-edge erosion problem is concerned, numerical studies can be of great utility if they can simulate the entire blade, its motion, and the erosion. This is not possible nor convenient experimentally, where tested samples may misrepresent the leading-edge erosion impact configuration.

In conclusion, the development of generalized erosion models is faced with many challenges including the coupled nature of the problem, selection of proper material parameters, representative damage mechanisms, and the role of surface topography in erosion initiation and progression. Fundamental experimental findings are needed to further iron out these issues so that more accurate erosion theories can be developed.

## 7. Erosion Behavior of Polymeric Materials

Although recent erosion studies focused more on metallic materials, there had been some efforts to understand the erosion behaviour of polymeric materials in the context of rain erosion that takes place in various components of aircrafts in the 1970s to 1990s [86,116,133]. In addition, there is a demand now to further understand the erosion behaviour of polymers, particularly elastomers and composites, as LEE of wind turbine blades is increasingly becoming a concern [149,150]. Unlike metals, polymers are very soft and experience severe erosion in subsonic conditions [116]. Also, the erosion mechanisms in metals are not similar to those in different classes of polymers. The ensuing discussion briefly outlines the current understanding of water droplet erosion behaviour of polymers.

### 7.1. Thermosetting and Thermoplastics

Early experiments [116,151] on a wide variety of polymeric materials showed that traditional thermosetting polymers such as epoxy, acrylic and polyester possess virtually no erosion resistance because of their inherent brittleness. Cracks appear first on the surface in the form of disconnected annular ring segments. Continued impingement leads to the intersection of these segments, which eventually results in chips of material removal [116]. 

Due to their superior ductility, thermoplastics possess higher erosion resistance compared to thermosets [22,116,152]. They usually fail in a ductile manner that is like those of ductile metals. Failure starts with initial surface depressions with upraised edges, which is facilitated by the fact that thermoplastics exhibit a sufficient plastic deformation without mass loss. Lateral jetting from the subsequent droplet impacts interacts with these edges leading to erosion pit nucleation. The surface depressions may represent a site of stress concentration but do not contribute to the erosion damage [76].

### 7.2. Polymer Composites

As mentioned, thermoplastics as bulk homogenous materials have superior erosion resistance compared to thermosets. However, according to several studies [116,151,153], the addition of fibre reinforcement to thermoplastic is detrimental to their erosion performance. This is because the fibres tend to pull out under the action of the repeated droplet impacts, which increases the overall mass loss. On the other hand, fibre reinforcement improves the erosion resistance of thermosetting composites because the fibres inhibit the massive chunking and breakage of the brittle thermoset resin. This is because the supposedly brittle resin holds the fibres in such a way that repetitive impacts of droplets and lateral outflow cause chipping and fracture of fibres rather than them pulling out. Also, thermosetting resin provides a discontinuous path for shock transmission through the material [153,154]. 

Fibre direction can also have an impact on the erosion behaviour of the composites. Gorham and Field [153] showed that a single droplet impact of unidirectional laminates can result in serious spall failure consisting of interconnected fractures and delamination. Whereas crossply laminates display less spalling because cracks are arrested at the interface with the subsequent orthogonal layer. However, this does not necessarily result in superior erosion performance of crossply laminates. A greater extent of damage in crossply laminates is often caused by stress-concentration effect as well as an impedance mismatch resulting from the difference in the direction of the layers [153]. 

Composites reinforced with random mat have shown less erosion resistance compared to uniform and directional reinforcement [153]. This is mainly because the irregular distribution of fibres results in lower fibre volume fraction and large resin-rich pockets which erode faster. Material loss can also be rapid when the short fibres of the mat are removed after the matrix failure. 

More analysis of the erosion behaviour of composite materials can be found in [116,152,154,155,156]. It can be said that modes of damage caused by rain erosion of composite materials are complex compared to homogenous polymers. This is because, given the inherent inhomogeneity of composite structures, the interaction between multiple impacts at a different position on a laminate can result in huge variability. Overall, fibre-reinforced polymer composites are intrinsically susceptible to severe erosion damage even at relatively low-impact speeds.

### 7.3. Elastomers

Erosion behaviour of elastomeric materials has been extensively studied in the past [22,116,152,157] as well as contemporary literature [158,159,160,161]. This is because, among different classes of polymer, elastomers were found to be the most erosion-resistant [6], and they are used to protect radomes and composite surfaces of aircraft against water droplet impact. The mechanism and properties with which elastomers can resist erosion are still unclear. It is known, however, that elastomers have two important characteristics that can be linked to their high erosion resistance; firstly, their low modulus and viscoelastic behaviour enable them to dissipate shockwave and the impact energy produced by droplet impact [86]. This essentially prevents impact pressure buildup, which results in stresses of low magnitudes inside the elastomers. This contrasts with hard and rigid solids, where the impact pressure and the resulting stresses are high, and the erosion resistance is stemmed from the ability of the material to withstand these high stresses rather than dissipating them in the first place. Secondly, elastomers have the ability to recover quickly from the impact of the droplet before the subsequent impact. This prevents the accumulation of stresses due to high-frequency impacts encountered in heavy rains [86].

The failure patterns of coatings made from elastomeric materials under water droplet impact have been historically studied by many researchers [116,152]. In most of the cases, failure of composite materials coated with elastomers takes place at the interface or in the composite underneath, while the surface of the elastomer remains intact [22,116]. Coating delamination occurs sometimes when one of the isolated surface flaws in elastomeric coating propagates after exposure to raindrop impacts. However, rarely that new pits form on the elastomeric surface due to subsonic droplets impact [152]. The next section outlines the use of elastomeric materials in leading edge protection coatings.

## 8. Leading Edge Protection Strategies

In general, operation and maintenance (O&M) of wind turbine amount to 20%–25% of the total cost per kWh produced over the lifetime of the unit [162]. This makes the repair of wind turbines very expensive, which usually includes erosion protection, repair of structural damages, and non-structural and cosmetic repair [162]. Among these repair issues, leading edge protection is the most important one. Hence, wind turbine original equipment manufacturers (OEMs), as well as coating suppliers, invest heavily in developing and implementing sound leading edge protection (LEP) measures to extend the life of the blade and combat against LEE. LEP primarily consists of applying surface coatings or tape to the leading edge of the turbine blade. Broadly, there are two surface coating techniques employed [6,159]:

- In-mould coatings: where application of the coating is integrated as a part of the manufacturing process of the blade. Usually, similar material to the composite matrix is used, such as epoxy or polyester. Such materials facilitate coating integration into the manufacturing process, which makes the techniques advantageous.
- Post-mould coatings: where coating is applied through painting or spraying. This allows the use of different flexible materials such as high erosion resistant elastomers.

**Figure 18:**
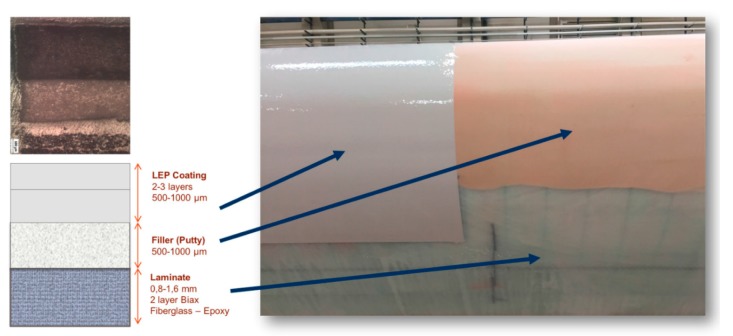
Example of multi-layered Leading Edge Protection System [159].

Post-mould coatings are used as LEP particularly in a location where leading-edge rain erosion is a threat. The coating may consist of one layer or it can be a multi-layered system, where one or more than a layer is applied between the laminate and the coating. An example of such a multi-layered protection system is shown in Figure 18. This may offer further damping to impact erosion. However, the presence of multiple interfaces may accelerate erosion due to delamination between layers. Moreover, post-mould coatings are sometimes used to repair damaged blades during service [159].

In terms of material, polyurethane is the most common elastomeric erosion resistant coating used for leading-edge protection. It was developed in the late 1960s to replace neoprene which had been in use since the early 1950s [151]. Thermoplastic polyurethanes (TPUs) now constitute the primary material for protective coatings against the erosion of the leading edge of wind turbine blades due to their high erosion resistance. Additives and reinforcements are usually added to further improve the erosion resistance of TPUs. These coatings are commonly referred to as ‘gelcoat’. The precise composition, additives involved, and the fabrication process of these coatings are primarily exercised by companies and not usually found in the open literature [6]. The current trend is the reinforcement of TPUs with nanomaterials [163], where improvement in erosion resistance is yet to be demonstrated. 

Although in most of the cases leading edge erosion is delayed using coating protection, these coatings cannot fully protect the blade composite material underneath. An earlier study by Schmitt [116] showed that although elastomers do not erode on the surface (in the studied speed range of 220 to 270 m/s), they suffer point failure at weak spots in the substrate underneath. They can also suffer from defects at the interface side due to the impact of erosion on the surface. This is mainly because water droplet impact involves stress waves that can travel through coatings and affect the interface. This may eventually lead to coating delamination even if the surface of the coating has not been eroded. Also, the application of elastomeric coatings and tape as leading edge protection solution has been proven to cause a drag increase of 5-15% compared to uncoated non-eroded blade [164]. 

Considering these issues, Bech et al. [12] recently proposed a different leading-edge protection strategy that is based on controlling wind turbine operation rather than the use of coatings. Bech et al. [12] postulated that the vast majority of the erosion damages takes place during extreme rain precipitation conditions, which cover a small fraction of the turbine life. Hence, by reducing tip speed during these events, leading edge erosion damage can be minimized [12]. However, the assumption that erosion only occurs in extreme rain conditions is questionable. Erosion is a complex phenomenon and once initiated, its progression can take place at normal rain condition. 

In summary, leading edge protection (LEP) techniques have demonstrated a limited success and they have yet to prove their durability in long-term. On-site repair, of the already installed turbines without protection, has varying quality. Efforts need to be dedicated to understanding erosion behaviour and role of interfaces present in LEP systems. 

## 9. Conclusions and Future Perspectives

This paper intended to highlight two important aspects of the water droplet impact erosion problem; its importance as it constitutes a concern for the wind energy industry as well as other industries; and the complexities involved in studying and tackling this erosion problem at fundamental levels. The ensuing discussion summarizes some of the important issues related to studying and modelling droplets impact erosion problem and possible future research fronts from the authors’ viewpoint.

**Mechanics and Damage Modes:** Fundamental studies devoted to understanding the physics and mechanics of water droplet erosion are needed. This is because other research aspects, such as protection, treatments, and modelling of WDE depend largely on the accurate understanding of erosion mechanics. The theory of impact pressure and stress waves has been the main explanation of the water droplet erosion phenomenon for more than four decades. However, how these waves eventually produce fracture, especially when the repetitive nature of the problem is considered, has not been made clear yet. Also, wave-defects interaction and the role of lateral jetting have not been well addressed. Moreover, surface fatigue, although likely to be involved in some materials, it is not the dominating erosion mechanism in others, as shown by experimental findings. Therefore, development of a comprehensive water droplet erosion theory that takes into considerations all these variables is needed. 

**Erosion Prediction Models:** Most of erosion prediction models in the literature are empirical models and valid for specific conditions. The success of fatigue-based erosion models is also limited, mainly due to the absence of accurate comprehensive erosion theory. Therefore, the development of more generalized erosion models is needed. This can begin with modifying the existing models (for example fatigue-based models) by incorporating a wide range of damage mechanisms as well as the contribution of the lateral jets and how they interact with the evolving surface features beyond the incubation period. Emphasis on erosion prediction models stems from the need for life prediction of components in real service applications. 

**Standardized Testing and Data Representation:** There is a strong need for a unified method of performing rain erosion tests. This is self-evident from the inability to quantitatively (and sometimes even qualitatively) correlate results from different erosion testing facilities throughout the literature even when ASTM G73 standard was followed. Central to erosion testing practices is the ability to measure and fully report the erosion parameters (speed, droplet size, frequency of impact, etc.). This has in some occasions been ignored and only weight loss for a certain period of time is provided. Equally important, is the need for a unified method of erosion test data representation. An attempt was made towards using impact energy as a principal parameter of erosion data representation that would allow accurate comparative studies. However, the success of impact energy was limited and further improvements are still needed.

**Correlation between Experimental Testing and Real In-Service Erosion:** Until now, accurate correlations between erosion performance at testing scale and real in-service performance have not been developed. This has, in some cases, rendered the accelerated experimental data useless to turbines manufacturers. The main obstacle of undertaking such research investigation has been the difficulty of accurately assessing the impact conditions (speed, droplet size, frequency, etc.) encountered in the real in-service application. This, however, seems to be possible to a reliable degree in case of leading-edge erosion of wind turbine blades. It is relatively less difficult to study the statistics and nature of raindrops interacting with the leading edge of the turbine blade in specific sites. This might be extremely difficult in other applications such as compressor of gas turbine and steam turbine blades, where the erosive environment or impact conditions are random. 

**Leading Edge Protection Materials:** Development of leading-edge protection materials is particularly important because the range of impact speeds involved in current wind turbine blade erosion is relatively small compared to other erosion applications. Consequently, a protection material with impact threshold velocity higher than that encountered in the blade while of course still having light weight can be sought. The problem, however, is that this practice is exercised mainly by industry and academic contribution is limited. Research in this front can begin with studying erosion behaviour of elastomeric materials and consequently, implementing necessary modifications. It is also important to understand the role of interfaces and adhesion strength on erosion damage behaviour.

Targeting these central issues may help in paving the way towards effectively combating WDE damage not only of the leading edge of the wind turbine, but in other applications as well.

## Figures and Tables

**Table 1:** Summary of the erosion conditions in some applications.

Application	Parts Affected	Impact Speed	Droplet Diameter	Ref
Steam Turbine	Blades of the low-pressure stage	400–900 m/s	50–400 µm	[17,18,33]
Gas Turbines	Compressor blades	100–600 m/s	200–600 µm	[21]
Wind turbine	the outer power-producing part	70–150 m/s	0.5–5 mm	[6,34]
Nuclear Power Plants	Cooling pipes	∼200 m/s	60–80 µm	[35]
Aero engine	fan blade	200–400 m/s	1–5 mm	[23]
Aircrafts	Rain erosion of different parts.	Civil airplanes ∼ 250 m/s Fighter Jets ∼ up to 5 Mach	1–5 mm	[25,36]
